# Insights into the Mechanism of Homeoviscous Adaptation to Low Temperature in Branched-Chain Fatty Acid-Containing Bacteria through Modeling FabH Kinetics from the Foodborne Pathogen *Listeria monocytogenes*

**DOI:** 10.3389/fmicb.2016.01386

**Published:** 2016-09-07

**Authors:** Lauren P. Saunders, Suranjana Sen, Brian J. Wilkinson, Craig Gatto

**Affiliations:** School of Biological Sciences, Illinois State UniversityNormal, IL, USA

**Keywords:** FabH, psychrotolerance, fatty acid biosynthesis, membrane fluidity, kinetic modeling, listeriosis, branched-chain carboxylic acids

## Abstract

The psychrotolerant foodborne pathogen *Listeria monocytogenes* withstands the stress of low temperatures and can proliferate in refrigerated food. Bacteria adapt to growth at low temperatures by increasing the production of fatty acids that increase membrane fluidity. The mechanism of homeoviscous increases in unsaturated fatty acid amounts in bacteria that predominantly contain straight-chain fatty acids is relatively well understood. By contrast the analogous mechanism in branched-chain fatty acid-containing bacteria, such as *L. monocytogenes*, is poorly understood. *L. monocytogenes* grows at low temperatures by altering its membrane composition to increase membrane fluidity, primarily by decreasing the length of fatty acid chains and increasing the anteiso to iso fatty acid ratio. FabH, the initiator of fatty acid biosynthesis, has been identified as the primary determinant of membrane fatty acid composition, but the extent of this effect has not been quantified. In this study, previously determined FabH steady-state parameters and substrate concentrations were used to calculate expected fatty acid compositions at 30°C and 10°C. FabH substrates 2-methylbutyryl-CoA, isobutyryl-CoA, and isovaleryl-CoA produce the primary fatty acids in *L. monocytogenes*, i.e., anteiso-odd, iso-even, and iso-odd fatty acids, respectively. *In vivo* concentrations of CoA derivatives were measured, but not all were resolved completely. In this case, estimates were calculated from overall fatty acid composition and FabH steady-state parameters. These relative substrate concentrations were used to calculate the expected fatty acid compositions at 10°C. Our model predicted a higher level of anteiso lipids at 10°C than was observed, indicative of an additional step beyond FabH influencing fatty acid composition at low temperatures. The potential for control of low temperature growth by feeding compounds that result in the production of butyryl-CoA, the precursor of SCFAs that rigidify the membrane and are incompatible with growth at low temperatures, is recognized.

## Introduction

The foodborne Gram-positive bacterial pathogen *Listeria monocytogenes* is the cause of the potentially serious disease listeriosis that is characterized by a high fatality rate. Detection of food contamination with *L. monocytogenes* continues to lead to large scale food product recalls. Recently, there has been a multistate outbreak of listeriosis linked to frozen vegetables (http://www.cdc.gov/listeria/outbreaks) that has resulted in an extensive recall of 358 consumer frozen vegetable and fruit products sold under 42 separate brands. Such outbreaks are very expensive, the costs of a 2008 outbreak in Canada being estimated at $242 million Canadian dollars (Thomas et al., 2015).

A critical aspect of *L. monocytogenes* in its role as a foodborne pathogen is its ability to grow at refrigeration temperatures and below to temperatures as low as −0.1°C (Walker et al., 1990). We have been interested in understanding the mechanisms underlying how *L. monocytogenes* copes with the stress of low temperatures over several years. A critical aspect of the psychrotolerance of the organism is to adjust its membrane fatty acid composition to maintain membrane fluidity. The fatty acids of *L. monocytogenes* are composed almost entirely of branched-chain fatty acids (BCFAs), with the three major fatty acids being the odd-numbered anteiso fatty acids anteiso C15:0 and anteiso C17:0, and odd-numbered iso fatty acid iso C15:0 (Annous et al., 1997; Nichols et al., 2002; Zhu et al., 2005). When the bacterium is grown at low temperatures the content of anteiso C15:0 rises markedly through a combination of reduction in fatty acid chain length and branching switching from iso to anteiso fatty acids (Annous et al., 1997; Nichols et al., 2002; Zhu et al., 2005). Anteiso fatty acids, which have a methyl branch on the antepenultimate carbon atom, disrupt the close packing of fatty acyl chains (Willecke and Pardee, 1971; Poger et al., 2014), resulting in increased membrane fluidity (Edgcomb et al., 2000), in what is termed homeoviscous adaptation (Sinensky, 1974).

The mechanisms involved in changes in fatty acid composition resulting in increased membrane fluidity are very different depending on whether the fatty acids of the bacterium are a mixture of straight-chain saturated fatty acids (SCFAs) and straight-chain unsaturated fatty acids (SCUFAs), or are predominately BCFAs. Species that contain primarily SCFAs and SCUFAs, which include many Gram-negative and some Gram-positive species, predominately alter fluidity via chain length and the ratio of SCFA to SCUFA (Zhang and Rock, 2008). In contrast, species with a high proportion of BCFAs, which are predominately Gram-positive species, alter chain length and the ratio of anteiso to iso fatty acids (Suutari and Laakso, 1994). Our work is involved in attempting to further understand the mechanisms underlying changes in fatty acid composition in *L. monocytogenes* that allow growth at low temperatures that may have applicability to BCFA-containing bacteria in general.

**Figure 1:**
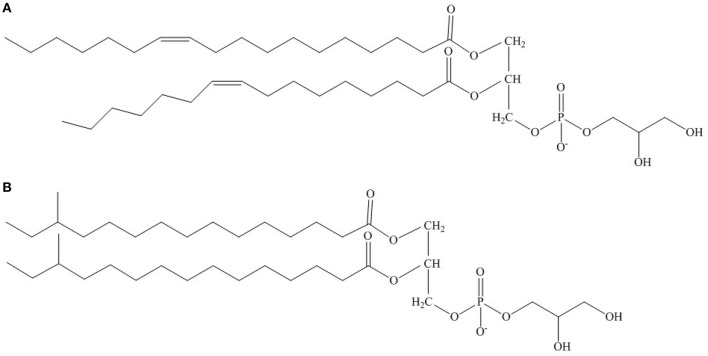
**Possible structure of phsophatidyl glycerol from (A) predominantly SCFA/SCUFA-containing bacteria and (B) predominantly BCFA-containing bacteria with particular reference to growth at low temperatures**. The fatty acids esterified at the 1- and 2-carbon positions are C18:1Δ11 and C16:1Δ9, respectively in **(A)** and anteiso C15:0 in both positions in **(B)**.

The mechanism by which SCFA- and SCUFA-containing bacteria increase the proportion of SCUFAs is understood in considerable detail in *Escherichia coli*. The major fatty acids in *E. coli* grown at 37°C are C16:0 (45%), C16:1Δ9 (35%), and C18:1Δ11 (18%) (Cronan and Rock, 2013). As growth temperature drops the percentage of C16:0 drops and C18:1Δ9 increases, thereby increasing the proportion of unsaturated fatty acids and membrane fluidity. The 1 position of *E. coli* phospholipids is primarily occupied by C16:0 and C18:1Δ11 and the C2 position by C16:1Δ9. At lower growth temperatures, C18:1Δ9 amounts increase at position 1 and C16:0 decreases (Figure 1). SCUFAs are produced by the activities of FabA and FabB. FabA dehydrates 3-hydroxyacyl-ACP to *trans*-2-enoyl-ACP during fatty acid elongation and at the 10-carbon stage *trans*-2-deconyl-ACP is isomerized to *cis*-3-decenoyl-ACP by FabA (Heath and Rock, 1996). *cis*-3-decenoyl-ACP is elongated by FabB rather than the FabF condensing enzyme to form C16:1-ACP, which is then elongated to 18:1-ACP prior to its incorporation into phospholipids by FabF. FabF is subject to thermal regulation and is responsible for increased C18:1Δ11 in cells grown at low temperatures (de Mendoza et al., 1983).

Homeoviscous adaptation in BCFA-containing bacteria appears to revolve around increasing the content of fatty acid anteiso C15:0 as exemplified by *L. monocytogenes* (Annous et al., 1997), *Bacillus subtilis* (Klein et al., 1999) and other BCFA-containing bacteria (Suutari and Laakso, 1994). Unsaturated fatty acids do not play a major role in homeoviscous lipid adaptation in BCFA-containing bacteria, which lack the *fabA* and *fabB* genes required for their synthesis (Lu et al., 2004). A cold-inducible system that introduces double bonds into existing phospholipids in *B. subtilis* is probably of minor significance (Cybulski et al., 2002) in long term cold adaptation. In BCFA-containing bacteria including *S. aureus*, anteiso C15:0 preferentially occupies position 2 in phospholipid molecules and anteiso C17:0 position 1 (Kaneda, 1991; Parsons et al., 2011). Fatty acid anteiso C15:0 rises to 65% or more of the total fatty acids in low temperature grown *L. monocytogenes* (Annous et al., 1997). It is likely that some of the anteiso C17:0 fatty acid on position 1 is replaced by anteiso C15:0 (Figure 1). Anteiso C15:0 then plays a similar role to the SCUFAs in fluidizing the membrane at low temperatures. We have much less knowledge of the mechanisms involved in increasing the proportion of anteiso fatty acids than SCUFAs.

The first condensation reaction in fatty acid biosynthesis is catalyzed by FabH, which plays a major role in determining the fatty acids produced by bacteria. The major fatty acids in *L. monocytogenes*, which contains almost exclusively BCFAs, are anteiso odd, iso odd, and iso even fatty acids, which are biosynthesized from 2-methylbutyryl-CoA (2MB-CoA), isovaleryl-CoA (IV-CoA), and isobutyryl-CoA (IB-CoA), respectively, produced through the activities of branched-chain amino acid transaminase and branched-chain α–keto acid dehydrogenase on the branched-chain amino acids isoleucine, leucine and valine, respectively. Interestingly, a non-native FabH will switch an organism's fatty acid composition to reflect that of the organism from which it originated (Choi et al., 2000; Li et al., 2005), showing that FabH substrate specificity plays a major role in fatty acid composition. We have examined the kinetics of *Lm*FabH at high and low temperatures and have provided evidence that the enzyme shows an increased preference for 2MB-CoA at 10°C compared to 30°C, which likely contributes to increased production of anteiso fatty acids at low temperatures (Singh et al., 2009).

Singh et al. (2009) measured *Lm*FabH kinetics and substrate concentrations at 30°C and 10°C to determine the role of FabH in the changes in relative fatty acid proportions at low temperatures in *L. monocytogenes*. These kinetic data describe the mechanism of FabH when only one substrate is present; however, endogenous substrate concentrations measured directly from *L. monocytogenes* show that all three branched-chain acyl Co-A derivatives are present under physiological conditions. Thus, competition exists between these substrates and therefore the *in vivo* kinetics must differ from the *in vitro* determined kinetic values obtained from individual substrates. In this paper, we extend the analysis in Singh et al. (2009) via calculations and simulations to estimate the *in vivo* kinetic parameters and preferences of *Lm*FabH. These *in vivo* substrate preferences can then be compared to the overall fatty acid composition of *L. monocytogenes*, which has been measured under a variety of conditions.

## Materials and methods

### Data modeling

**Figure 2:**
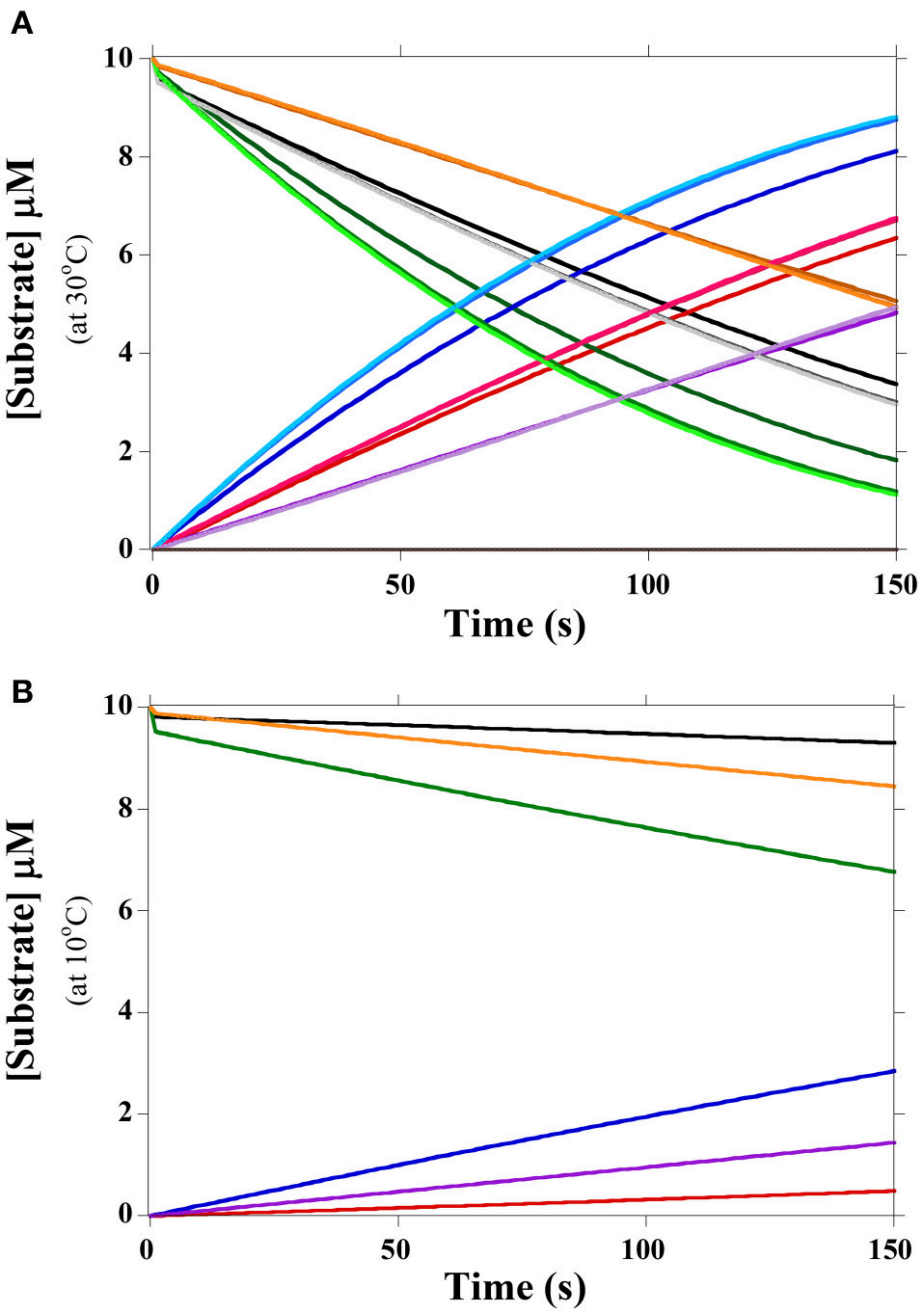
**Rates of FabH activity with branched substrates at 30°C and 10°C. (A)** Rates of substrate hydrolysis and product formation with equal substrate concentrations (10 μM) at 30°C. IB-CoA is orange and its product purple; IV-CoA is black and its product red; 2MB-CoA is green and its product blue. Forward rates for the pre-*k*_cat_ equilibrium varied from 0.1 s^−1^ to 10 s^−1^ and are color coded from light to dark. *Lm*FabH concentration was 1 μM. Ac-CoA substrate is not shown as it is off scale for the duration of the experiment; its product is gray (at 0 μM). **(B)** Rates of substrate hydrolysis and product formation with equal substrate concentrations (10 μM) at 10°C. IB-CoA is orange and its product purple; IV-CoA is black and its product red; 2MB-CoA is green and its product blue. Forward rates for the pre-*k*_cat_ equilibrium were set to 10 s^−1^. *Lm*FabH concentration was 1 μM.

Data were modeled according to the Briggs-Haldane mechanism for all substrates simultaneously using Scheme 1:

$$\begin{array}{l} \begin{array}{c} ES_{1}\\ \;k_{1}↑↓k_{-1} \end{array}\overset{k_{2}}{�}E+P_{1}\\ \begin{array}{c} E+S_{1}+S_{2}+S_{3}\\ k_{-5}↑↓k_{5} \end{array}\overset{k_{3}}{\underset{k_{-3}}{⇄}}ES_{2}\;\overset{k_{4}}{�}\;E+P_{2}\text{ Scheme }1\\ \;ES_{3}\;\overset{k_{6}}{�}E+P_{3} \end{array}$$

in which FabH is *E*; *S* and *P* refer to the different substrates and products, respectively, and *k*_1_/*k*_−1_, *k*_3_/*k*_−3_, *k*_5_/*k*_−5_ refer to the forward and reverse rates associated with the *K*_M_. The values *k*_2_, *k*_4_, and *k*_6_ are the *k*_cat_ rate constants for chemical conversion of the various substrates to their corresponding products. As the concentration of malonyl-acyl carrier protein, i.e., the second substrate, was constant for all experiments (Singh et al., 2009), it was incorporated into the *K*_M_ and *k*_cat_ values, reducing the kinetic mechanism to the Briggs-Haldane equation. Reaction rates were determined from Singh et al. (2009) with a reverse catalytic rate of zero. Rates for the initial equilibrium were modeled from the *K*_M_ by setting the forward reaction rate to a constant for all substrates and varying the reverse reaction rate to account for *K*_M_ value. Changes to the forward reaction rate did not significantly affect results (Figure 2B). Not all acyl-CoA substrates could be separated via HPLC in Singh et al. (2009); thus, temperatures were compared both under equal substrate conditions and under the values reported for *in vivo* conditions.

Composite steady-state parameters were calculated from the steady-state parameters of the individual substrates in Singh et al. (2009) using the equation:

(1) $$\frac{V_{S1}}{\left[E\right]}=\frac{V_{\max}[S_{1}]}{\left[S_{1}]+K_{M1}(1+\frac{\left[S_{2}\right]}{K_{M2}}+\frac{\left[S_{3}\right]}{K_{M3}}\right)}$$

in which *V*_*S*1_ is the velocity of hydrolysis of substrate 1 and *S* and *K*_M_ correspond to the concentration and measured *K*_M_ of the species denoted in the subscript, respectively. This equation is modified from the equation for two competitive inhibitors (Segel, 1975) by replacing the inhibitors with substrates 2 and 3 and using the Briggs-Haldane rather than the Michaelis-Menten constants.

### Determination of fatty acid composition

*L. monocytogenes* strain 10403S was grown in Brain Heart Infusion (BHI) media at 30°C, under conditions identical to those in Sen et al. (2015). *L. monocytogenes* was grown concurrently in unsupplemented BHI and BHI supplemented with increasing amounts of the lipid precursors 2-methylbutyrate (2MB), isovalerate (IV), isobutyrate (IB), and butyrate (B), that act as precursors for biosynthesis of odd-numbered anteiso, odd-numbered iso, even-numbered iso BCFAs and even-numbered SCFAs, respectively (Julotok et al., 2010). *L. monocytogenes* cultures were harvested in exponential phase and the fatty acid compositions were determined as described by Zhu et al. (2005).

## Results

FabH can utilize both straight- (acetyl-CoA; Ac-CoA) and branched- (IV-CoA, 2MB-CoA, and IB-CoA) chain substrates; however, *Lm*FabH greatly prefers branched-chain substrates. The specific activity of FabH with Ac-CoA is greater than 10-fold lower than with the three branched substrates (Singh et al., 2009). Simulations based on Scheme 1 show that negligible amounts of Ac-CoA are utilized when all four substrates are present in equal concentrations (Figure 2). The presence of Ac-CoA therefore has nearly no impact on FabH substrate utilization and was not analyzed in subsequent simulations. FabH's low affinity for Ac-CoA agrees well with the native fatty acid composition of *L. monocytogenes*, as there are negligible amounts of SCFAs in its membrane. As Ac-CoA is present at relatively high concentrations (18.0 μM at 30°C), lack of this precursor is not preventing *L. monocytogenes* from making SCFAs. Rather, our data suggest that FabH substrate selectivity results in the lack of SCFAs.

Simulations show FabH prefers 2MB-CoA as a substrate at both 30°C and 10°C with equal substrate concentrations of 2MB-CoA, IV-CoA, IB-CoA, and Ac-CoA (Figure 2, Table 1). 2MB-CoA produces anteiso fatty acids, the predominant membrane fatty acids in *L. monocytogenes*. The next best substrates only produce ~75% (IV-CoA) and ~50% (IB-CoA) of the 2MB-CoA product at 30°C and 10°C, respectively (Figure 2). Interestingly, IV-CoA is the second best utilized substrate at 30°C, but is not preferred over IB-CoA at 10°C. As IV-CoA is converted into iso-odd fatty acids, this modeled decrease in preference likely reveals the reason for observed lower proportion of these fatty acids at low temperatures (Annous et al., 1997; Nichols et al., 2002; Zhu et al., 2005).

**Table 1:** **Normalized amount of product produced at various temperatures and substrate concentrations by FabH activity**.

**Temperature (°C)**	**Substrate**	**Independent calculations (Singh et al., 2009) (%)**	**Dependent calculations (3 substrate model) (%)**	**Dependent calculations (2 substrate model) (%)**
30	IV-CoA	29.8	33.3	n/a
	2MB-CoA	53.2	51.4	80.5
	IB-CoA	17.0	15.3	19.6
10	IV-CoA	14.3	9.0	n/a
	2MB-CoA	57.1	63.7	71.1
	IB-CoA	28.6	27.4	28.9

Calculations were done using Equation (1), with no substrate inhibition, equal amounts of 2MB-CoA and IV-CoA (i.e., 2.85 μM 2MB-CoA and 2.85 μM IV-CoA at 30°C), and the extreme case in which only 2MB-CoA is present with no IV-CoA.

In the simplest model for the determination of fatty acid composition, only FabH contributes to fatty acid composition and that composition reflects the substrate concentrations and FabH's enzymatic properties. This hypothesis is described by Scheme 1. To determine the validity of this hypothesis, substrate concentrations are needed for FabH's three main substrates. While Singh et al. (2009) measured endogenous substrate concentrations *in vivo*, not all substrates were resolved. For instance, butyryl-CoA (B-CoA) could not be separated from IB-CoA, and 2MB-CoA could not be separated from IV-CoA. Substrate concentrations were therefore varied within the range of values measured in Singh et al. (2009) to see if the data from the substrate concentrations, enzymatic parameters, and fatty acid composition could be described by the model (Scheme 1). As SCFAs (i.e., the products from B-CoA) are present only in low amounts in *L. monocytogenes*, B-CoA is not expected to contribute significantly to the fatty acid profile. Thus, the *in vivo* amount of IB-CoA can be estimated directly from the membrane fatty acid composition.

**Figure 3:**
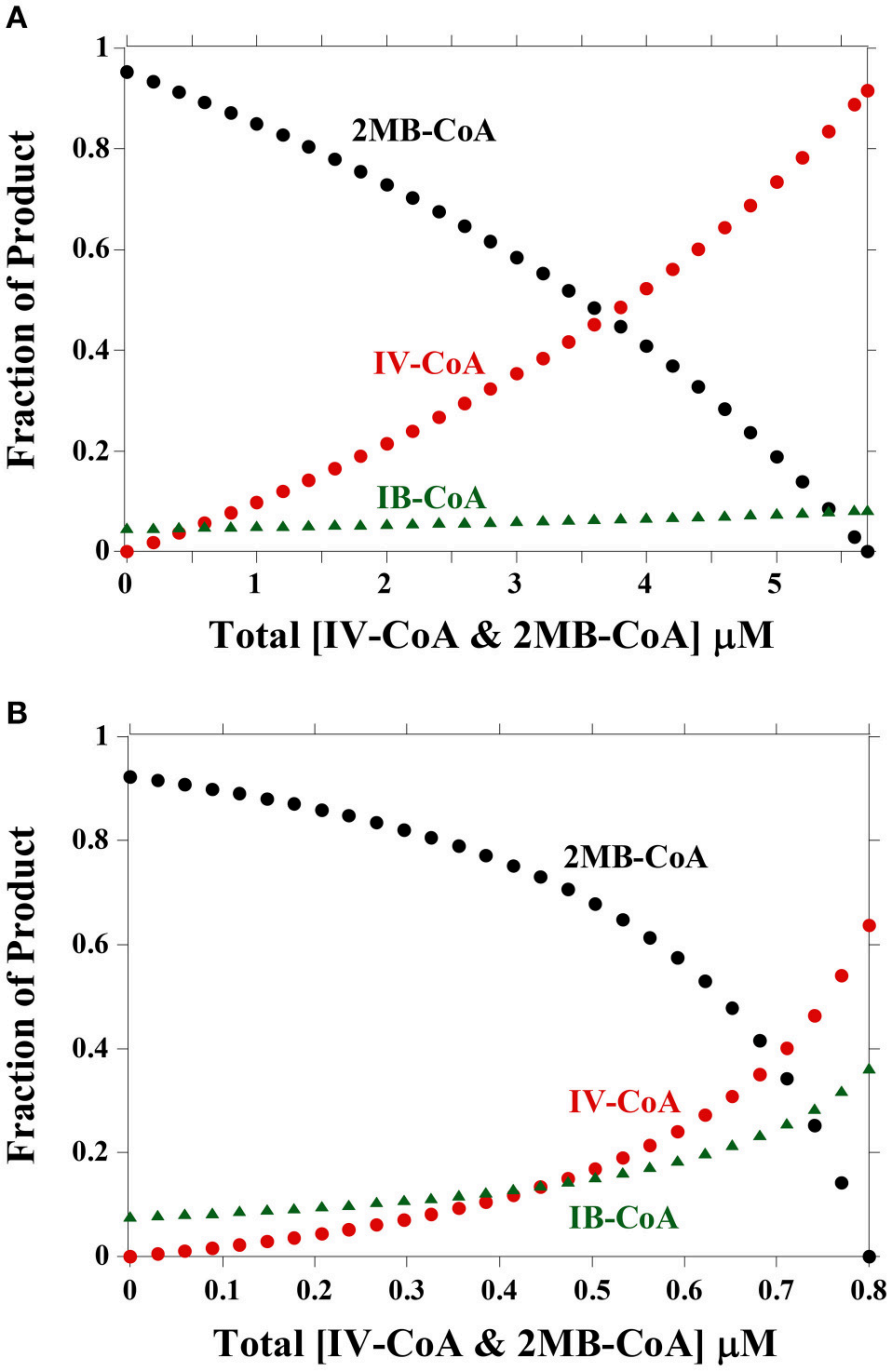
**Percent product produced by FabH with varying concentrations of IV-CoA and 2MB-CoA. (A)** Fraction of product based on concentrations and kinetic parameters at 30°C. Product amounts are calculated from Equation (1). Total IV-CoA and 2MB-CoA concentration is 5.7 μM, thus the [2MB-CoA] = 5.7μM – [IV-CoA]. IB-CoA concentration is 0.9 μM. **(B)** Fraction of product based on concentrations and kinetic parameters at 10°C. Product amounts are calculated from Equation (1). Total IV and 2MB concentration is 0.8 μM, thus the [2MB-CoA] = 0.8μM – [IV-CoA]. IB-CoA concentration is 0.149 μM.

Figure 3 shows a model depicting the expected relative concentrations of fatty acids for varying IV-CoA and 2MB-CoA concentrations. As IB-CoA concentration is not changing, the amount of iso-even fatty acids produced changes much less than either anteiso- or iso-odd fatty acids. An IB-CoA concentration of 0.9 μM would result in the observed amount of ~5% even-numbered iso-fatty acids at 30°C (Figure 3A). At 10°C, an equivalent ratio of IB-CoA:B-CoA was used to model lipid composition which resulted in slightly higher iso-even fatty acid concentrations of ~10% (Figure 3B). This is a higher concentration of iso-even fatty acids than is observed experimentally (Annous et al., 1997; Nichols et al., 2002; Zhu et al., 2005). Therefore, while the model can describe experimental data obtained at 30°C, the model cannot describe the fatty acid composition experimentally observed at both temperatures without changes in the relative substrate concentrations. Specifically, iso-even fatty acid levels have never been reported as high as 10% as predicted by the model at 10°C (Figure 3B); thus, the easiest resolution to this discrepancy is that *in vivo* an additional step regulates either the supply of acyl-CoA precursors to FabH or the incorporation of iso-even fatty acids into the lipid bilayer.

As for the 2MB-CoA and IV-CoA concentrations, in BHI media at 30°C, the proportions of anteiso and iso odd fatty acids are 81.5 and 13.8%, respectively, giving a product ratio of 5.9 (Zhu et al., 2005). Modeling at 30°C suggests this product ratio of fatty acids occurs at 1.4 μM IV-CoA and 4.3 μM 2MB-CoA, a substrate ratio of 3.0. Thus, there is good agreement between the model and measured fatty acid content at 30°C. If changes in FabH's substrate preferences alone are responsible for the differences in the lipid profile, then the 10°C fatty acid ratio (i.e., 7.5) should correspond to the same substrate ratio (3.0). However, at 10°C a substrate ratio of 3.0 (i.e., 0.2 μM IV-CoA, 0.6 μM 2MB-CoA) produces a calculated fatty acid product ratio of 19.5, which greatly exceeds what is observed (Annous et al., 1997; Nichols et al., 2002; Zhu et al., 2005). This dichotomy between the model and the actual fatty acid composition measured suggests that FabH, although critical in determining final membrane fatty acid content, may not be the sole regulatory step involved. The simplest explanation for the substantial overestimated anteiso fatty acid content predicted by the model is that an additional regulatory step alters either the concentration of acyl-CoA precursors, or a later regulatory step regulates the amount of anteiso fatty acids that get incorporated into the lipid bilayer. As with IB-CoA, the model describes the data at 30°C, but cannot describe the data at both temperatures, and thus another step must be involved in determining fatty acid composition.

To further investigate the effects of FabH, *L. monocytogenes* was cultured in medium supplemented with fatty acid precursors and the effects on fatty acid composition were observed. Concentrations of fatty acid precursors were small enough that no effect on bacterial growth was observed (data not shown). The amount of exogenously added short-chain carboxylic acid made available to FabH as the corresponding CoA derivative is uncertain which hampers our calculations. Thus, we necessarily assume that the partitioning of the substrate is a consistent percentage of the exogenously added precursor which becomes available to FabH. This percentage of available substrate was added to the model as an additional variable in our calculations (i.e., 2MB + *x*%^*^ [exogenous 2MB]) and simulations of fatty acid composition were run using this equation.

**Figure 4:**
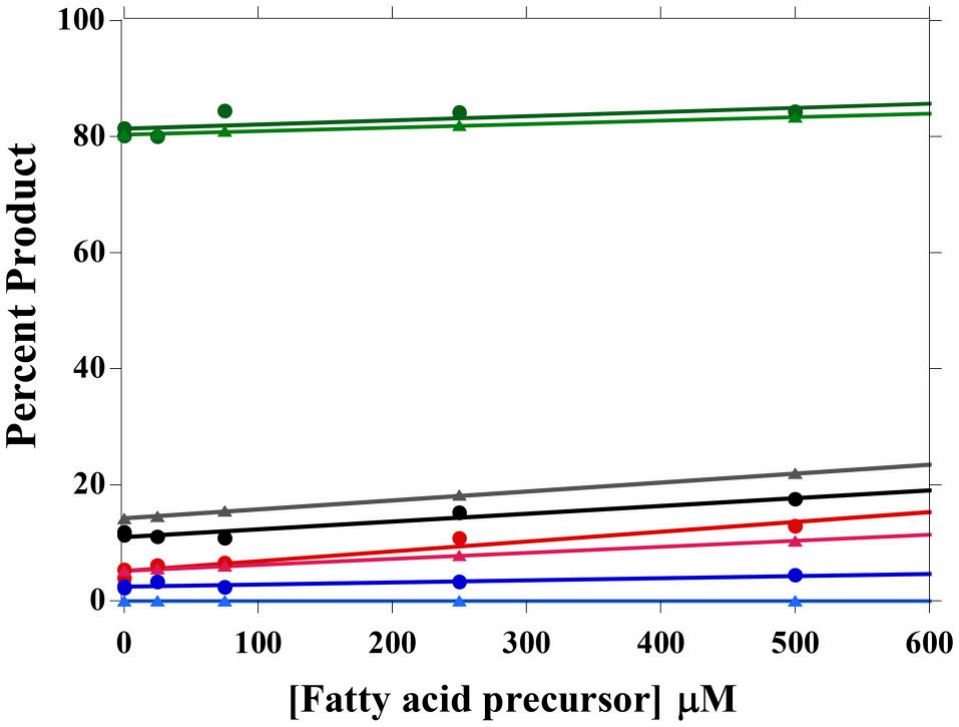
**Membrane fatty acid composition changes upon fatty acid precursor addition**. 0, 25, 75, 250, and 500 uM of 2MB (green), IV (black), IB (red), and B (blue) were added to *L. monocytogenes* cultures and the fatty acid compositions were determined. The increase in the fatty acid precursor's fatty acid product was measured and modeled using FabH substrate preferences (triangles, lighter colors).

The effect of precursor addition (i.e., 2MB, IV, IB, and B) on the fatty acid composition of *L. monocytogenes* was measured, and the amount of available substrate was calculated from fatty acid composition. Based on our model, 0.2% of the exogenously added substrate is available to FabH under steady-state conditions, and substrate identity does not affect substrate availability (Figure 4). The increase in the associated product was measured for each fatty acid precursor added, and the data are well described by the model (Figure 4). As the kinetic parameters for B were not measured in Singh et al. (2009), the Ac-CoA parameters were instead used in the fitting. B-CoA appears to be a better FabH substrate than Ac-CoA, as more of it is incorporated into the membrane fatty acids than predicted from Ac-CoA kinetic parameters. 2MB-CoA, IV-CoA, and IB-CoA percentages are well described by FabH preference.

## Discussion

FabH plays an integral role in determining membrane fatty acid composition in *L. monocytogenes*, as it does for other bacteria (Choi et al., 2000; Cronan, 2003). In this report, we modeled the activity of FabH at both 30°C and 10°C and found a qualitative explanation for the fatty acid profile change that occurs in this organism between high and low temperatures. This provides confidence that FabH is one of the enzymes that promotes growth at low temperatures for *L. monocytogenes* via increases in membrane fluidity. However, our model does not quantitatively mimic the exact distribution fractions of fatty acids at both temperatures, suggesting that another enzyme combines with FabH to produce the final membrane composition in *L. monocytogenes*.

In order to model fatty acid composition with additional substrates in the growth media, one must have an estimate of the amount of available cellular substrate. Millimolar quantities of fatty acid precursors are usually added to produce fatty acid composition changes (Julotok et al., 2010). This implies that only low levels of fatty acid precursors are converted into fatty acids, consistent with our finding that only ~0.2% of exogenously supplemented precursors was available to FabH. It is not yet known whether this limitation is due to slow precursor uptake by the cell or another slow enzymatic step in producing the CoA derivatives of the various precursors. All four exogenous precursors appear to be equally available to FabH, as the percentage of available substrate was the same for all four substrates. This percentage would be expected to change if the substrate concentrations were tightly regulated, so it appears that there is little regulation of substrate concentrations at 30°C.

An increase in anteiso fatty acids at the expense of iso-odd fatty acids at lower temperatures is clearly shown in our model; however, the extent of replacement predicted by the model exceeds that seen *in vivo*. Our model assumes no change in the relative amounts of IV-CoA and 2MB-CoA under different temperature conditions; however, the concentrations of IV-CoA and 2MB-CoA were not separable in Singh et al. (2009). Thus, the deviation from the model may reflect alterations in relative substrate amounts, which would be compatible both with the data and our model. Alternatively, an upstream step from FabH that leads to the production of the CoA substrates may also be temperature dependent. The IV-CoA pool must increase from ~1/3 at 30°C to ~2/3 of the IV-CoA / 2MB-CoA pool at 10°C to fit the ratio seen in the fatty acid composition data. This requires a significant change in an earlier step in fatty acid production, and thus an additional enzyme that helps control fatty acid composition in *L. monocytogenes*. If substrate concentrations are not temperature dependent, a downstream step from FabH could selectively prefer iso-odd fatty acids at lower temperatures to regulate a possible overproduction of anteiso fatty acid precursors by the enzyme. The lower than predicted incorporation of anteiso fatty acids could be an adaptation for a rapid response to temperature change; when the temperature decreases, e.g., the cells need additional anteiso fatty acids, and FabH's preference for 2MB-CoA could be modulated via a feedback mechanism once the appropriate level of anteiso fatty acids are synthesized.

## Conclusion

No other studies have measured endogenous FabH substrate concentrations, so there is no basis for comparison to determine how well FabH substrate preference in other organisms compares to final fatty acid composition. In this study, fatty acid composition was calculated from FabH substrate preferences and composition, and the differences between this model and the actual fatty acid compositions of *L. monocytogenes* were compared. The data presented here suggest that FabH is the primary controller of fatty acid composition in *L. monocytogenes*. FabH preference can be used to predict fatty acid composition at 30°C with and without added substrates, and qualitatively predict temperature induced changes in fatty acid composition. However, an additional control step beyond FabH alone is required to adequately predict fatty acid composition changes at lower temperatures.

Further in the type II fatty acid biosynthesis pathway is the rate limiting enzyme FabI (enoyl-ACP reductase). Schiebel et al. (2012) reported the ratio of specificity constants of FabI from *S. aureus* (a BCFA-containing gram-positive bacterium) was 1:24:1, straight: iso: anteiso. This is a potential candidate for a further control point that could be temperature regulated. FabH substrate preference plays a significant role in *L. monocytogenes* survival at low temperatures, and methods can be devised to exploit this step to control *L. monocytogenes* growth. Similarly, identifying the enzymatic processes in addition to FabH that control the levels of lipid bilayer BCFA content may also reveal potential targets for controlling growth of this organism at low temperatures. For example, fatty acid composition can be modified by manipulation of precursor concentrations (Julotok et al., 2010) and encouraging the biosynthesis of SCFAs that are derived from B-CoA would decrease the growth of *L. monocytogenes* at low temperatures.

## Author contributions

CG: Designed experiments, evaluated and interpreted data, contributed extensively to the writing of the manuscript, supported the science with grant funds. BW: Designed experiments, evaluated and interpreted data, contributed extensively to the writing of the manuscript, supported the science with grant funds. LS: Developed the kinetic model, ran simulations, evaluated and interpreted data, contributed extensively to the writing of the manuscript. SS: Grew strains of *L. monocytogenes*, carried out fatty acid analyses, helped with data interpretation.

## Funding

This work was supported by grant R15-AI099977 from the National Institute of Health to BW and R15-GM61583 to CG.

### Conflict of interest statement

The authors declare that the research was conducted in the absence of any commercial or financial relationships that could be construed as a potential conflict of interest.
